# Genetic characterization of Chikungunya virus 2009 isolates from South India

**DOI:** 10.6026/97320630014106

**Published:** 2018-03-31

**Authors:** Gopalsamy Sarangan, Seema A Nayar, Monika Mani, Sudharsana Sundarrajan, Sathish Sankar, Gunasekaran Palani, Gracy Fathima Selvaraj, Jayachandran Damodharan, Karuppaiah Muthumani, Padma Srikanth

**Affiliations:** 1 Department of Microbiology, Sri Ramachandra Medical College and Research Institute, Chennai, India; 2 Department of Microbiology, Trivandrum Medical College, Trivandrum, India; 3 Department of Biotechnology, Vellore Institute of Technology, Vellore, India; 4 Sri Sakthi Amma Institute of Biomedical Research Sri Narayani Hospital and Research Centre, Sripuram, India; 5 Department of Virology, King Institute of Preventive Medicine, India; 6 Saveetha Institute of Medical and Technical Sciences, Chennai, India; 7 The Wistar Institute, Philadelphia, USA

**Keywords:** Chikungunya virus, partial E2 gene, RT-PCR, substitutions, selection pressure

## Abstract

Chikungunya Virus (CHIKV) is a single stranded positive sense enveloped RNA virus. Re-emergence of CHIKV caused a massive outbreak with severe clinical manifestation affecting multiple organs. The genetic diversity of CHIKV, which caused recurring outbreaks in India, was studied. Blood samples were collected from suspected human cases of CHIKV infection in Chennai, Tamil Nadu and three Northern districts of Kerala in Southern India during the CHIKV outbreak in 2009. A partial E2 gene segment was amplified by RT-PCR. Among 119 samples 37 samples were positive for CHIKV by RT-PCR. Phylogenetic analysis revealed that the isolated sequences belonged to Indian Ocean Lineage (IOL) of ECSA genotype. The mutational analysis revealed the presence of substitutions such as S299N, T312M, A344T, S375T, V386G, W339R and S375P in the current study. In addition, a novel mutation V386G was observed in all the sequences. Two isolates found with unique substitutions W339R and S375P are reported. The structural analysis of the wild type and mutant proteins revealed that the structural changes are accompanied by modification in the intraprotein interactions.

## Background

CHIKV is a single stranded positive-sense RNA virus belonging to alphavirus genus of the Togoviridae family. The 12kb viral genome encodes four non-structural proteins (nsP1, nsP2, nsP3 and nsP4), a core protein and three structural proteins (E1, E2 and E3, E1 and E2 are connected by a short 6K region) [1]. Various gene mutations in structural and non-structural protein coding regions may contribute to infectivity and enhanced virulence rate of the virus [2]. Several mutations in the protein coding regions were detected during the 2006 epidemic in Reunion Island [3]. A point mutation in E1 protein (A226V) altered vector specificity, which resulted in the re-emergence of CHIKV and has been detected among CHIKV isolates during 2007 epidemic in the Indian subcontinent including Kerala [4]. E2 and E1 act as trimers of heterodimers (E2-E1) on the viral particle surface. E1 is a class II viral protein that mediates the fusion of viral membrane with host cell membrane under acidic pH. E1 homotrimer attaches to the host membrane and forms a hairpin loop; E2 most likely mediates cell attachment. The E2 protein has domains called A, B and C. Domain B and A has significant role in receptor binding. Domain B is located at the membrane distal part and forms the tip of E2. Domain A is located at the center and domain C is close to the viral membrane. Domain C is a transmembrane protein that attaches to the hairpin loop of E1 and aids in the viral entry [5]. Therefore, it is of interest to explore the gene mutations of the highly conserved C domain of E2 protein during the 2009 outbreak to understand the impact of domain C in viral entry. The potential selection pressure sites were analysed for their influence on evolution. The effect of mutations on protein structures provides valuable insights to functional consequences.

## Methodology

### Ethics statement

Institutional ethical committee approval was obtained to carry out the study (IEC-NI/09/APR/09/11). The study participants were recruited after obtaining a written informed consent.

### Sample collection

Blood samples were collected from the suspected CHIKV cases from Kozhikode and Kannur districts of Kerala and a tertiary care centre in Chennai, Tamil Nadu during October-November, 2009. Blood samples were collected in suitable appropriately labeled vacutainer tubes (Beckton Dickinson, USA) and transported to the laboratory at 4°C. A detailed proforma on clinical symptoms and duration of illness was also obtained. Plasma was separated and stored in aliquots at -80°C.

### RNA extraction and RT-PCR

The viral RNA was extracted from the plasma using QIAamp Viral RNA Mini kit (Qiagen, Hilden, Germany) according to manufacturer's protocol. The partial E2 gene of CHIKV was amplified by RT-PCR using Qiagen One step RT-PCR kit (Qiagen, Hilden, Germany) on a thermo cycler (Applied Biosystems, Veriti, USA) using the primers forward: TATCCTGACCACCCAACGCTCC and reverse: ACATGCACATCCCACCTGCC [6]. The amplicons were verified for the size (305 bp) using agarose gel electrophoresis.

### Partial E2 gene Sequencing

DNA Sequencing was performed in our laboratory. The amplified DNA was mixed with RNase free water and made up to the volume of 100μl and filtered under vaccum in the Multiscreen HTS PCR plate (Millipore, USA). Big dye terminator cycle sequencing ready reaction kit (Applied Biosystems, USA) was used for sequencing with specific primers targeting partial E2 region [6]. The sequencing mixture was again purified by Montage SEQ 96 filtration (Millipore, USA). DNA sequencing was performed on ABI genetic analyzer 3730 (Applied Biosystems, USA). The sequences (37) were deposited in GenBank database and the accession numbers are KJ577651-KJ577660, KC977313- KC977322 and KM275635-KM275651.

### Phylogenetic analysis

From GenBank database, (n=360 sequences) CHIKV sequences were retrieved (DOA: 24/02/2016) and analysed along with study sequence. E2 gene was taken for analysis along with our sequences. Sequences were categorized based on the year of isolation, continent and genotypes. Likelihood mapping analysis was done to avoid the false conclusion about evolutionary relationships among strains using MEGA 7 [7]. Consensus was made for each category using codon code aligner (Tamura, Stecher et al. 2013).

### Selection pressure analysis

The nucleotide sequences (n=37) were aligned using HyPhy package implemented in Datamonkey server [8] and checked for redundancy. The potential recombination in the dataset was screened using GARD method. The selection pressure in the nucleotide sequence alignment was assessed using Parris method. The specific site selection on the gene was analysed using Single likelihood ancestor counting (SLAC), fixed effects likelihood (FEL), random effects likelihood (REL), MEME and fast-unconstrained bayesian approximation (FUBAR) algorithm. Positive selection was defined as p-value ≤ 0.1 for SLAC, FEL and Posterior probability ≥ 0.9 for FUBAR.

### E2 protein structural analysis

The secondary structure of the protein was predicted by PDBSUM [9]. The entire protein structure was unavailable in PDB; an ab-initio structure prediction was carried out using ITASSER [10]. The best model was selected using confidence score (C-score) and validated using Ramachandran plot predicted by SAVES server [11]. The substitutions reported in all the study isolates were modelled using Swiss PDB viewer [12]. All the structures were optimized using Gromacs 4.5.6 [13]. The structural consequences were investigated by comparing the wild type and mutant protein structures. The structures were visualized using PyMol. Changes in the intra-molecular interactions were analysed using PIC server [14].

## Results & Discussion

### Demography and clinical features of the patients

All 119 samples were collected from the study participants with acute onset of fever along with joint pain, myalgia and headache. Of these 119 samples, CHIKV infection was confirmed in 37 study participants by RT-PCR. Since all the samples were collected during acute phase illness (<5 days), Chikungunya IgM was not performed. Age of the participant's age ranged between 7 and 75 years (mean 35.7 years, p < 0.0001). We compared the prevalence rate based on the age of the patients by grouping them into four categories as; < 14, 15-24, 25-50 and 51-75 years. Significant detection of CHIKV was observed in the adults who are 25 to 50 years old (p < 0.05). The male female ratio was 1:1.43. Table 1 lists the demographic profile of 37 participants positive for CHIKV by RT-PCR. The clinical features fever (100%) and severe joint pain (97.29%) were most frequent joint inflammation (7%), maculo-papular rash on trunk and limbs (8.10%) and headache (10.81%) were also observed.

### Nucleotide sequencing and Phylogenetic analysis

The partial E2 protein-coding region showed changes at twenty nucleotide positions. Among them, seven unique nucleotide changes were consistent in all the sequences (Table 2). These nucleotide substitutions were common among studied isolates [15].

**Figure 1:**
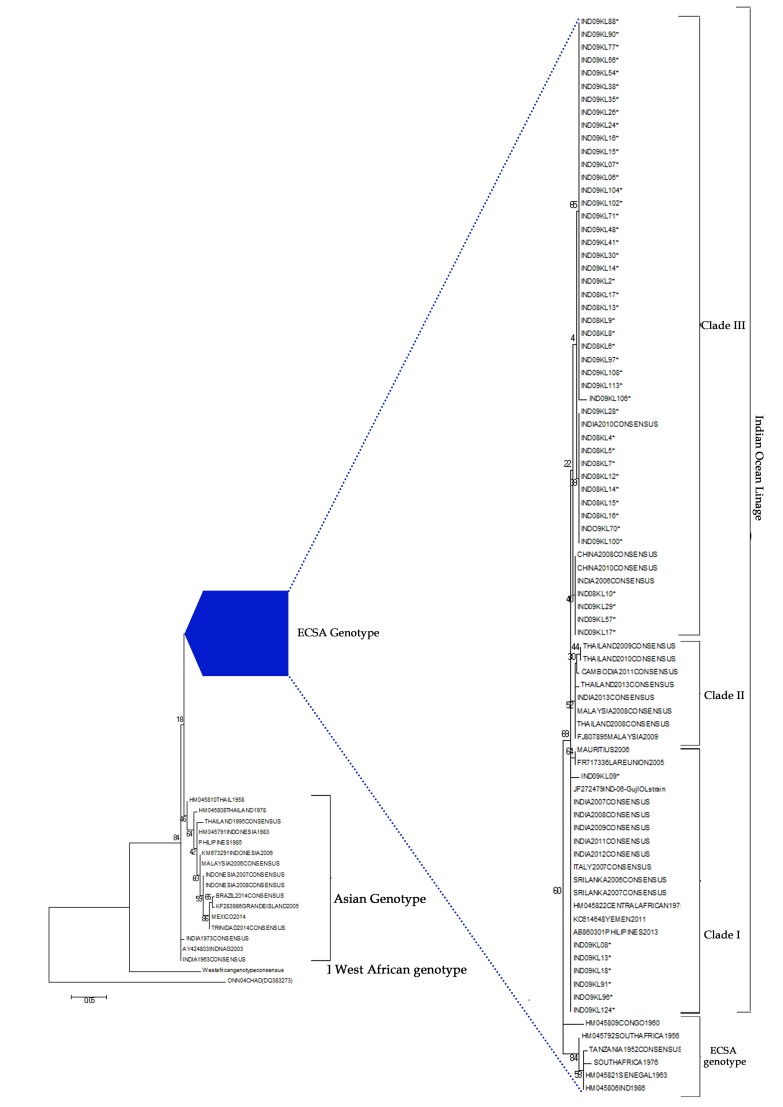
Phylogenetic tree of CHIKV isolates and with the sequences derived from GenBank database generated using a maximum likely hood on the partial sequence of E2 gene (305bp). Bootstrap analysis was performed with 1000 replicates to determine confidence values on the clades within trees. The S27 strain (AF369024) prototype strain and O'nyong nyong (out group) were used in the tree.

Phylogenetic tree constructed using geographic sequences of different genotypes has shown that all the sequences belonged to ECSA genotype (Figure 1). ECSA genotype group was rooted with strains isolated from Congo during 1960. Three clusters were observed in the ECSA lineage. Majority (n=24) of the sequences were found in Clade III along with sequences isolated from Asian countries. Rest of the sequences (n=13) were found in-group I with the sequences of Central Africa, Italy and South East Asian countries. None of the sequences were found in clade II (which denotes sequences from South East Asia and India). The strains isolated from India after 2012 were found in-group II. This may be due to the amino acid divergence. Nucleotide divergence was found to be 5.2%, 4.8% and 5.5% among Clade I, Clade II and Clade III respectively based on the CHIKV prototype strain. No specific genetic diversity was noted in the consensus sequence of 2007-2010 isolated from different periods. Indian 2010 isolates were closely associated with these sequences. Branch length of 2011-2016 consensus sequence was more when compared to all other sequences due to nucleotide and amino acid divergence. Year wise analysis showed that isolates from 2006 to 2009 were found in the same cluster (Figure not shown).

The most recent common ancestor (MRCA) in our analysis was La reunion 2005 strain. Indian Ocean strain outbreak started from Kenya in 2004 and emerged from ECSA genotype. Phylogenetic analysis showed two different lineages for Indian Ocean and Indian subcontinent as previously described [16]. Positionspecific alignments were performed to examine the extent of amino acid conservation in the E2 gene to assess the spatial dynamics of the substitutions at specific positions. Sequences of study isolates were compared with S27 African and Indian Oceanic Lineage (IOL) prototype sequences. Five site specific mutations; S299N (S27), T312M, A344T, S375T, A386G (IOL) and V386G (S27) were observed. The substitutions in E2 gene at positions 312, 344 and 375 have already been observed and reported among the Indian isolates [17]. The substitutions in E2 gene at 299 and 386 were noted in 29 and 23 study isolates, respectively. The unique substitutions: A386G (IOL), V386G (S27), W339R (KC977314) and H349P (KJ57760) were reported for the first time in the C-domain of E2 protein.

### Selection pressure analysis

The GARD analysis identified no possible recombination in the sequence alignments. The results of the PARRIS analysis indicated no evidence of positive selection in the sequence alignment. Of the four methods used to test for positive selection, FEL identified five negative selection sites at 22, 29, 83, 84,100 and one positive selection site. SLAC method identified one negative selection site at codon number 29. MEME identified codon 35 and 45 as positive sites with evidence of episodic diversifying selection. FUBAR identified codon 22 and 100 as sites with evidence of pervasive purifying selection (Table 3). Based on the prediction of protein secondary structure, codon 35 is located on the thirtieth beta turn, codon 45 on the tenth beta-bulge and codon 78 on the sixth helix. Codons 35, 45 and 78, that are under episodic positive selection experience purifying selection for their evolution [18]. Mutations at such sites may face transient positive selection, which indicates adaptive evolution towards new vector adaptability, cell tropism of vector and human.

### Effect of mutation on protein structure

**Figure 2:**
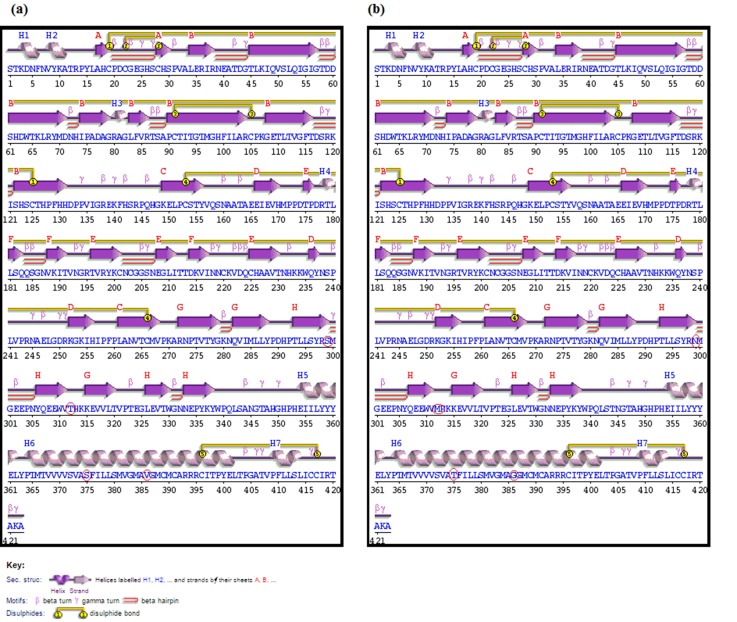
The secondary structure of the (a) Wild type and (b) Mutant proteins. The amino acid substitutions are highlighted.

The four substitutions that were prevalent in all the sequences were compared with the wild type to assess the changes in the protein structure. The secondary structure analysis of the wild type and mutant partial E2 proteins revealed that no major structural change had been enforced; however, the S299N substitution lead to loss of gamma turns and the mutated residue (Asparagine) is found within a beta-strand (Figure 2).

**Figure 3:**
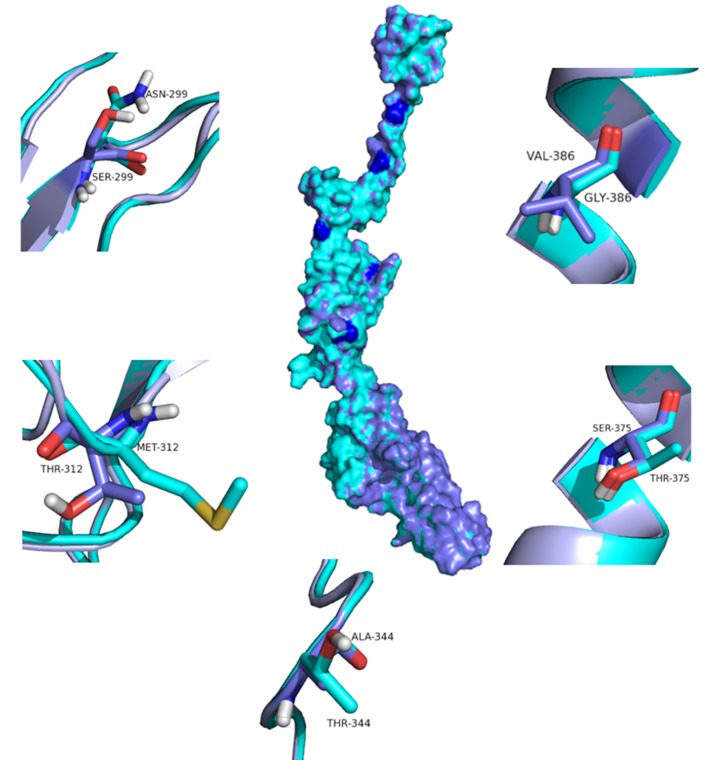
Mapping the positions of selected major mutations identified in CHIKV isolates on to the structural regions of the corresponding protein. The Wild type (violet) and mutant (cyan) are superimposed and represented as surface.

The C-score of the protein structures predicted by I-Tasser ranged between -2.35 and 1.02. The model 5 with a C-score of 1.02 was selected for further analysis. The quality of the best model was verified using Ramachandran plot. The predicted protein model had 75.9% of the residues in the most favoured region, 23.9% of the residues in additionally allowed region and 0.3% of residues in the disallowed region validating the quality of the model generated. A structural deviation of 0.019 Å was observed when the wild type and mutant proteins were superimposed (Figure 3). The intra-molecular interaction analysis revealed various changes in the main chain hydrogen bonds, main chain - side chain hydrogen bonds, side chain-chain hydrogen bonds and hydrophobic interactions (Table 4 and 5). Possibly, this may alter the interaction of E2 with other proteins, particularly with cellular receptors, and may impact on tissue tropism [18]. A study proved that mutation in E2 gene leads to more severe inflammation and damage in the tissues distal to the site of the inoculation in the mice that were inoculated with CHIKV strain with mutation in E2 and in 3'UTR. Mutation in E2 along with 3'UTR enhances dissemination of virus to different organs with increases in titre of virus [19]. Since the recent outbreak is evidenced with severe joint pain in majority of the patients, it may be due to amino acid substitution in viral proteins, which leads to enhancement of host cell binding affinity. The comparison of the intramolecular analysis showed deviations in the bond formed by the residues. In addition, many new interactions were gained and few interactions were lost during the substitution process. The changes in the protein structure in the mutant may be compensated by gain or loss of new bonds to maintain their structural integrity leading to their adaptability and evolution.

## Conclusion

All sequences showed V386G substitution and we hypothesize that this substitution cause conformation changes in the protein leading to better affinity towards human host tissues. More studies are required to understand the effects of novel substitutions in the E2 region. Our study results along with published reports [20, 21, 22, 23] indicate that there is a persistent change in the genome of CHIKV strains. Amino acid substitutions, which lead to structural changes of the proteins, lead to enhanced pathogenicity and virulence. Further studies are required to correlate the amino acid substitutions with disease severity, vector adaptation, host-cell interaction and drug affinity.

## Figures and Tables

**Table 1:** Demographic and clinical features associated with CHIKV positive

Demographic and clinical factors	CHIKV positive cases (n=37)
Age (years) mean + SD	40.51+ 15.94
Male: Female ratio	15:22
Fever	37
Joint Pain	36
Joint Inflammation	7
Maculo-papular rash on trunk and limbs	3

**Table 2:** Nucleotide and amino acid variations of E2 gene in the study samples in comparison with ECSA lineage and other Indian samples are summarized below

Nucleotide position	Amino acid variation	Samples				
ECSA	Indian samples	All study samples	KC977314	KJ57760
9437*	S299N	G	A/G	A		
9476*	T312M	C	T/C	T		
9556*	W339R	T	T/C	.	A	
9571*	A344T	G	A	A		
9587*	H349P	A	.	.		C
9664*	S375T	T	A/T	A		
9698*	V386A	T	C/T	G		

**Table 3:** Amino acid Sites of CHIKV structural protein under significant positive and purifying selection

Method	Codon no. in Structural Protein	Positively selected amino acid site	Negatively selected amino acid site	P-value	Posterior probability
FEL	78	365-T		0.078	
	22		309-E	0.028	
	29		316-E	0.069	
	83		370-V	0.075	
	84		371-V	0.075	
	100		387-G	0.002	
SLAC	29		316-E	0.062	
MEME	35	322-P		0.088	
	45	332-N		0.046	
FUBAR	22		309-E		0.937
	100		387-G		0.973

**Table 4:** Intra-protein interactions of the wildtype and mutant proteins

Proteins	Interactions	DONOR			ACCEPTOR			Parameters			
		Position	Residue	Atom	Position	Residue	Atom	Dd-a	Dh-a	A(d-H-N)	A(a-O=C)
Wild Type	Intra-protein Main Chain-Main Chain Hydrogen Bonds	299	S	N	304	P	O	3	2.37	119.6	130.12
		299	S	N	305	N	O	2.88	1.92	157.67	135.67
		301	G	N	299	S	O	2.88	2.14	130.46	100.7
		346	G	N	344	A	O	3.33	3.11	93.79	76.23
		375	S	N	371	V	O	2.98	2.08	148.42	154.42
		375	S	N	372	S	O	3.04	2.45	117.65	123.07
		375	S	N	373	V	O	3.33	3.39	77.95	78.72
		377	I	N	375	S	O	3.43	3.48	79.11	76.08
		378	L	N	375	S	O	3.26	2.83	106.73	112.91
		379	L	N	375	S	O	2.79	1.84	156.86	162.97
		386	V	N	382	V	O	2.87	1.91	166.51	161.61
		386	V	N	383	G	O	3.39	2.92	110.71	112.7
Mutant		299	N	N	304	P	O	2.74	1.8	154.3	151.06
		301	G	N	299	N	O	2.88	1.95	153.16	86.78
		291	H	N	312	M	O	3.44	2.75	127.41	152
		346	G	N	344	T	O	3.27	3.03	95.07	75.32
		375	T	N	371	V	O	2.97	2.06	150.42	157.64
		375	T	N	372	S	O	3.04	2.45	117.54	123.52
		375	T	N	373	V	O	3.33	3.4	77.72	78.59
		377	I	N	375	T	O	3.38	3.43	79.06	77.38
		378	L	N	375	T	O	3.22	2.8	106.48	115.24
		379	L	N	375	T	O	2.77	1.82	158.11	165.34
		386	G	N	382	V	O	2.85	1.9	159.02	158.96
		386	G	N	383	G	O	3.24	2.76	110.16	112.16
		386	G	N	384	M	O	3.45	3.57	75.06	76.52
		388	M	N	386	G	O	3.49	3.64	73.18	75.13
		389	C	N	386	G	O	3.25	2.75	111.61	110.44
		390	M	N	386	G	O	2.94	1.99	161.69	161.18
Wildtype	Intra-protein Main Chain-Side Chain Hydrogen Bonds	299	S	OG	303	E	O	2.71	9.99	999.99	170.58
		312	T	OG1	291	H	O	3.28	9.99	999.99	74.22
		375	S	OG	371	V	O	2.69	9.99	999.99	140.97
		389	C	N	386	V	O	3.29	2.82	110.08	104.54
		390	M	N	386	V	O	2.92	1.96	160.67	157.37
Mutant		299	N	ND2	303	E	O	2.85	3.39	51.34	127.33
		299	N	ND2	303	E	O	2.85	1.87	154.41	127.33
		299	N	OD1	325	G	O	3.48	3.99	54.4	144.62
		299	N	OD1	325	G	O	3.48	2.41	173.85	144.62
		301	G	N	299	N	OD1	3.46	3.33	88.94	89.1
		375	T	OG1	371	V	O	2.65	9.99	999.99	138.55
Wildtype	Intra-protein Side Chain-Side Chain Hydrogen Bonds	-									
Mutant		299	N	OD1	324	E	OE1	3.42	2.51	142.92	999.99
		299	N	OD1	324	E	OE1	3.42	3.94	54.2	999.99
		299	N	OD1	324	E	OE2	3.21	2.36	135.32	999.99
		299	N	OD1	324	E	OE2	3.21	4.12	27.61	999.99
		299	N	ND2	324	E	OE2	2.9	1.91	156.28	999.99
		299	N	ND2	324	E	OE2	2.9	3.68	36.77	999.99
		324	E	OE1	299	N	OD1	3.42	3.59	72.24	999.99
		324	E	OE1	299	N	OD1	3.42	3.94	53	999.99
		324	E	OE2	299	N	OD1	3.21	3.39	70.97	999.99
		324	E	OE2	299	N	OD1	3.21	3.58	61.21	999.99
		324	E	OE2	299	N	ND2	2.9	2.58	96.37	999.99
		324	E	OE2	299	N	ND2	2.9	3.03	73.2	999.99

**Table 5:** Hydrophobic interactions of the wild type and mutant proteins

Proteins	Position	Residue	Position	Residue
Wild type	382	V	386	V
Mutant	292	P	312	M
	310	T	312	M
